# Superior anti-tumor efficacy of diisopropylamine dichloroacetate compared with dichloroacetate in a subcutaneous transplantation breast tumor model

**DOI:** 10.18632/oncotarget.11609

**Published:** 2016-08-25

**Authors:** Lei Su, Hailin Zhang, Chen Yan, Aiping Chen, Gang Meng, Jiwu Wei, Decai Yu, Yitao Ding

**Affiliations:** ^1^ Department of Hepatobiliary Surgery, Affiliated Drum Tower Hospital of Nanjing University Medical School, Nanjing 210008, P.R. of China; ^2^ Jiangsu Key Laboratory of Molecular Medicine, Medical School and the State Key Laboratory of Pharmaceutical Biotechnology, Nanjing University, Nanjing 210008, P.R. of China

**Keywords:** diisopropylamine dichloroacetate, dichloroacetate, subcutaneous transplantation breast tumor model, MDA-MB-231 cell line

## Abstract

Dichloroacetate (DCA), an inhibitor of pyruvate dehydrogenase kinase, has anti-tumor properties in various carcinoma models. Diisopropylamine dichloroacetate (DADA), an over-the-counter drug for chronic liver disease, is a derivative of DCA. To date, few studies have evaluated the anticancer potential of DADA in breast cancer. In this study, MDA-MB-231 cells, a breast adenocarcinoma cell line, were used in *in vitro* and *in vivo* experiments to evaluate the anti-tumor efficacy of DADA and DCA. The half maximal inhibitory concentration (IC_50_) of DADA (7.1 ± 1.1 mmol/L) against MDA-MB-231 cells was significantly lower than that of DCA (15.6 ± 2.0 mmol/L); 100 mg/kg (0.0004 mol/kg) DADA was better than 100 mg/kg (0.0008 mol/kg) DCA at suppressing the growth of subcutaneous transplantation breast tumor at the same dose after 24 days intervention. Histological examination showed that both DCA and DADA interventions led to necrosis, inflammation, and fibrosis of tumor tissue in a mouse subcutaneous transplantation breast tumor model. DADA treatment inhibited Ki67 expression in tumor tissue. In vitro experiments showed that DADA could inhibit lactic acid production and glucose uptake in MDA-MB-231 cells at 10 mmol/L and these effects were stronger than DCA. DADA administration also induced complete autophagy during early treatment stages and incomplete autophagy and cell death at later treatment stages. In conclusion, DADA showed better anti-tumor efficacy than DCA in a breast cancer model.

## INTRODUCTION

Breast cancer develops from breast cells [1]. The most common type of breast cancer is ductal carcinoma, which begins in the cells of the ducts [2]. Breast cancer can also begin in lobule cells or in other breast tissues [2]. Breast cancer in women is a major public health problem in both developed and developing countries; over 508,000 women died from it in 2011 [3]. Breast cancer is typically treated with surgery followed by chemotherapy, radiation therapy, or both [2, 4]. The outcome depends on various factors, including the cancer classification, cancer stage, and patient age [5]. Although multidisciplinary approaches, including hormone-blocking therapy for hormone-receptor-positive cancers and monoclonal antibodies to treat certain cases of metastatic cancer, have improved the survival rate in the developed world, breast cancer remains a major challenge for women's health worldwide, especially in developing countries [3].

Like all other cancers, breast cancer occurs when breast cells uncontrollably divide and evade cell death [6]. Breast cancer occurrence is generally determined by the interaction between environmental (external) factors and genetic susceptibility [7]. An example of genetic susceptibility to breast cancer includes mutations in the breast cancer 1 (*BRCA1*) or *BRCA2* gene, which results in a lifetime risk of breast cancer of between 60% and 85% [8]. However, only 5 to 10% of cases are due to inherited genes [9]. Many other risk factors, including age, obesity, smoking, lack of physical exercise, alcohol consumption, hormone replacement therapy during menopause, ionizing radiation, early age at first menstruation, and having children late or not at all, were found to increase the risk of breast cancer [10, 11]. In general, our knowledge on the pathophysiology of breast cancer is lacking. Therefore, extensive research is needed to improve breast cancer management.

A low level of glycolysis and oxidation of pyruvate occurs in the mitochondria of most normal cells [12]. This process is aerobic, and is termed oxidative phosphorylation [12]. In contrast to normal cells, most cancer cells predominantly produce energy by increasing the rate of glycolysis and lactic acid fermentation in the cytosol to support rapid cell division and growth [13]. The glycolytic rates of rapidly growing malignant tumor cells can be 200 times higher than that in normal cells [14]. This reprogramming of energy metabolism in cancer cells is collectively known as the Warburg effect, and is a valid therapeutic strategy for cancer treatment [13, 14].

Another cell mechanism altered in cancer cells is autophagy, which is a natural destructive mechanism that disassembles unnecessary or dysfunctional cellular components [15]. During autophagy, targeted cytoplasmic constituents are isolated within a double-membraned vesicle known as an autophagosome [16]. The autophagosome then fuses with a lysosome, and the contents are degraded and recycled through a regulated process [15–19]. Two proteins involved in autophagy, cytosolic microtubule-associated protein 1 light chain 3 (LC3) and sequestosome 1 (p62), are highly conserved and play important roles in key stages of autophagy [15–19]. In the LC3 processing pathway, LC3-I is conjugated to phosphatidylethanolamine (PE) to become LC3-II via activating enzyme autophagy-related (*Atg*)7 genes and the conjugating enzyme Atg3 [15–19]. p62 can deliver selective autophagic cargo for degradation by binding directly to LC3 and gamma-aminobutyric acid (GABA) receptor-associated proteins [15–19]. The p62 protein is itself degraded by autophagy; p62 accumulates when autophagy is inhibited, while p62 quantities decrease when autophagy is induced [20].

The role of autophagy in cancer has been well researched [15–20], yet two opposing views exist. Autophagy can contribute to cancer by promoting survival of tumor cells that have been starved due to excessive energy demand or through the degradation of apoptotic mediators [21]. In these cases, autophagy inhibitors, such as chloroquine, could complement antineoplastic drugs [22]. However, recent research suggests that autophagy could suppress tumor growth by reducing necrosis and chronic inflammation [23]. In addition, a single allele deletion of Beclin-1, an Atg, is common in human breast, ovarian, and prostate cancers, suggesting that autophagy may have tumor suppressor properties [24].

The tricarboxylic acid (TCA) cycle is a major link among carbohydrate, lipid, and amino acid metabolism [25]. Dichloroacetate (DCA) is a pyruvate dehydrogenase kinase inhibitor, which can preferentially divert glucose metabolism from glycolysis towards oxidative phosphorylation through the activation of pyruvate dehydrogenase [26, 27]. DCA has been shown to have anti-tumor properties in various carcinoma models, and its clinical application is currently being investigated in trials [26, 27]. A DCA homolog, diisopropylamine dichloroacetate (DADA), also known as vitamin B15, improves energy metabolism in hepatocytes and has been used for decades to treat chronic liver disease [26, 27]. Recent studies have shown that DADA has potential therapeutic effects on colorectal cancer [26, 27]. However, existing research has not fully determined the anti-tumor mechanisms of DADA or DCA. In addition, to date, few studies have evaluated the anticancer potential of DADA in breast cancer. In this report, the effect of DADA and DCA on lactic acid production and glucose uptake, autophagy, tumor growth, and the expression of nuclear antigen Ki67 were examined in a breast adenocarcinoma cell line and in subcutaneous transplantation breast tumors.

## RESULTS

### DADA inhibits proliferation in MDA-MB-231 cells

**Figure 1:**
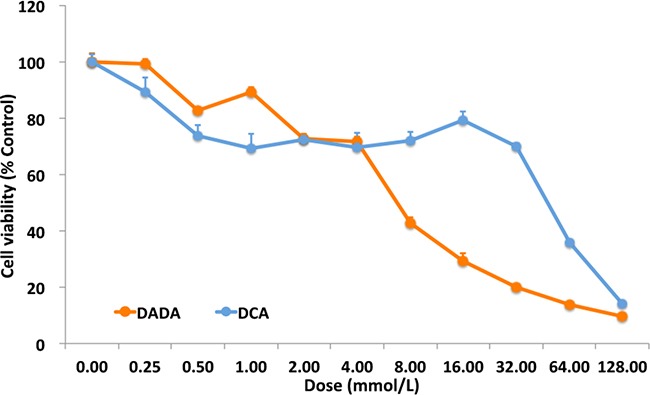
MTT assay and crystal violet staining Serial dilutions of DADA and DCA solutions, from 128.00 mM to 0.25 mM, were added to MDA-MB-231 cells. After 48-h incubation, cell viability was checked by MTT assay. The viability of cells with no treatment was set as 100%.

Our preliminary *in vitro* and *in vivo* experiments showed that DADA had a broad spectrum of anti-tumor capabilities. To systematically study the anti-tumor effect of DADA, MDA-MB-231 cells were used in the following *in vitro* and *in vivo* experiments. First, the *in vitro* anti-tumor capabilities of DADA and DCA were evaluated using the IC_50_ method. Serially diluted DADA and DCA solutions were added to MDA-MB-231 cells and incubated for 48 h. Cell viability was examined using MTT assays. As shown in Figure 1A, cell viability decreased in a concentration-dependent manner when treated with DADA and DCA. The IC_50_ of DADA was 7.1 ± 1.1 mmol/L, which was significantly lower than the IC_50_ of DCA (15.6 ± 2.0 mmol/L) (*P* < 0.05).

### DADA inhibits tumor growth in a subcutaneous transplantation tumor model

**Figure 2:**
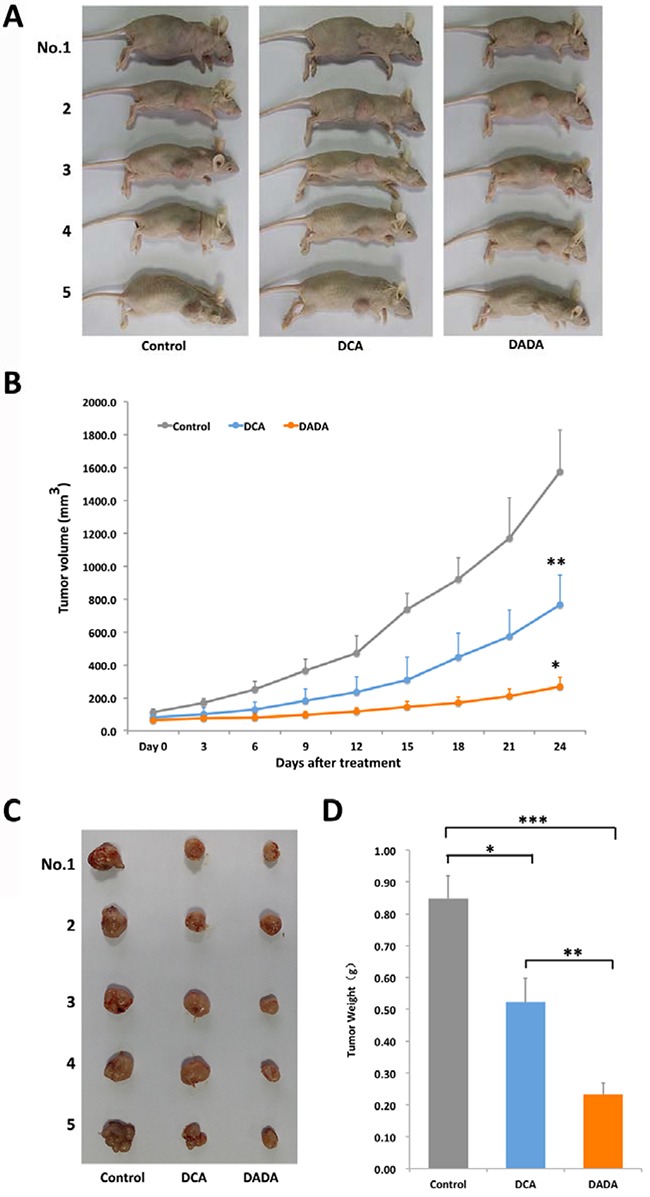
DADA inhibits tumor growth in a subcutaneous transplantation tumor model **A.** A subcutaneous transplantation tumor model in nude BALB/c mice was established using the human breast cancer cell line MDA-MB-231. The mice were divided into control, DCA, and DADA groups with 5 mice per group. **B.** The growth curve of tumor volumes: *, *P* < 0.05 compared with the DCA-treated group; **, *P* < 0.05 compared with the control. **C.** Subcutaneous transplantation tumor isolates. D, The average tumor weights of control, DCA-, and DADA-treated mice: *, **, and *** indicate *P* < 0.05.

To evaluate if DADA possessed *in vivo* anti-tumor capabilities, we established a subcutaneous transplantation tumor model in nude BALB/c mice using MDA-MB-231 cells. Once the subcutaneously transplanted tumors had grown to 100–150 mm^3^, the mice were divided into 3 groups (5 in each group): control, DADA, and DCA (Figure 2A). The mouse groups were lavaged once a day with 100 μL 0.9% saline (control group), 100 mg/kg (0.0004 mol/kg) DADA (DADA-treated group), or 100 mg/kg (0.0008 mol/kg) DCA (DCA-treated group) for 24 d. Tumor volume was measured every 3 d. As shown in Figure 2B, the tumor volumes of the control, DADA-, and DCA-treated mice increased from 82.5 ± 21.6, 80.4 ± 16.8, and 81.2 ± 25.5 mm^3^ on Day 0 to 1,575.7 ± 253.3, 765.9 ± 180.6, and 267.6 ± 57.2 mm^3^ on Day 24, respectively. The tumor volumes of DCA-treated mice (765.9 ± 180.6 mm^3^) were significantly smaller than those in control mice (1,575.7 ± 253.3 mm^3^) (*P* < 0.05); the tumor volumes of DADA-treated mice (267.6 ± 57.2 mm^3^) were significantly smaller than those in DCA-treated mice (765.9 ± 180.6 mm^3^) (*P* < 0.05). On Day 24, all tumors were isolated for analysis. The smallest tumors were found in the DADA-treated group, with average tumor weights of 0.23 ± 0.04, 0.52 ± 0.08, and 0.85 ± 0.07 g for DADA, DCA, and control mice, respectively. Both DCA and DADA treatments significantly inhibited tumor growth compared with the control (Figure 2C and 2D). In conclusion, the *in vivo* experiments showed that 0.0004 mol/kg DADA had stronger anti-tumor capabilities than did 0.0008 mol/kg DCA.

### Low Ki67 expression occurs in tumor tissues after DADA treatment

**Figure 3:**
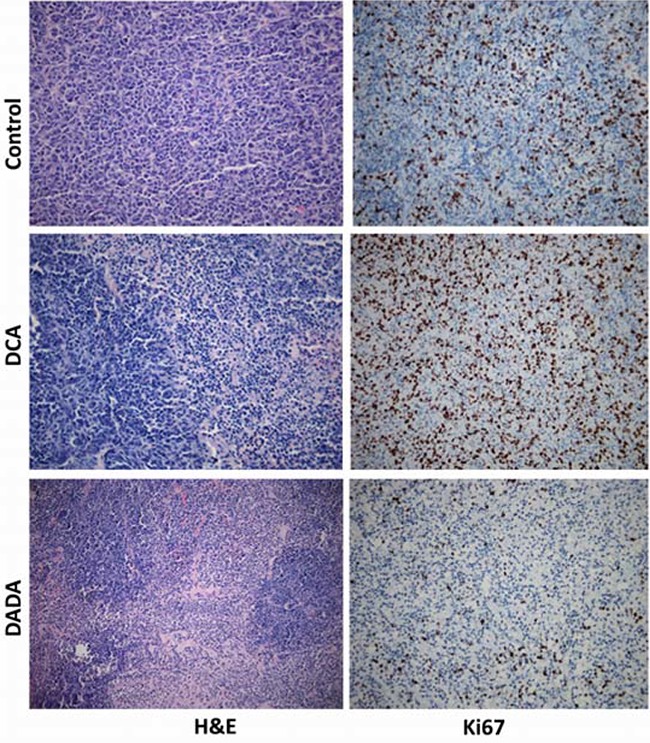
Histopathological and immunohistochemical examination On Day 24, all tumors were isolated for histopathological examination with H&E and Ki67 staining assays.

On Day 24, all tumors were isolated for histopathological examination. H&E staining showed that subcutaneous transplantation tumor growth was vigorous in control mice, while tumor necrosis, fibrosis, and inflammation were clearly observed in DADA- and DCA-treated mice (Figure 3, left). We then evaluated Ki67 expression in tumors isolated from control, DCA-, and DADA-treated mice using immunohistochemical staining. Ki67 was highly expressed in the tumors of control and DCA-treated mice, while its expression was greatly reduced in the tumors DADA-treated mice (Figure 3, right). In conclusion, DADA intervention reduced Ki67 expression.

### DADA inhibits lactic acid production and reduces glucose uptake in MDA-MB-231 cells

**Figure 4:**
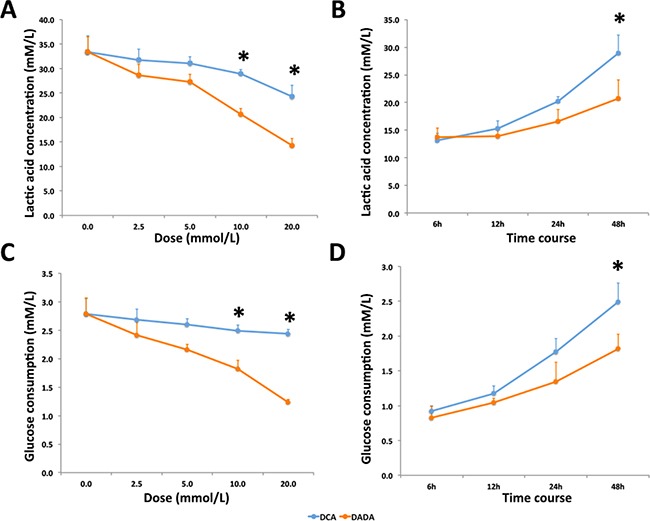
DADA inhibits lactic acid production and glucose uptake in MDA-MB-231 cells **A.** Cells were treated with 0.0 to 20.0 mM DADA or DCA, and the lactic acid concentrations were measured after 48-h incubation; * indicates *P* < 0.05. **B.** DADA and DCA concentrations were set at 10 mmol/L, and lactic acid concentrations were measured at 6, 12, 24, and 48 h; * indicates *P* < 0.05. **C.** Cells were treated with 0.0 to 20.0 mmol/L DADA or DCA, and glucose concentrations were measured after 48 h; * indicates P < 0.05. **D.** DADA or DCA concentrations were set at 10 mmol/L, and glucose concentrations were measured at 6, 12, 24, and 48 h; * indicates *P* < 0.05.

DCA is an inhibitor of pyruvate dehydrogenase kinase, and can increase aerobic oxidation of glucose and reduce lactic acid production in various tumor cells. DCA mainly affects tumor cells and has little effect on the metabolism of normal cells. Because the effective component of DADA is DCA, we investigated whether DADA could also inhibit lactic acid production and reduce glucose uptake in MDA-MB-231 cells. Cells were incubated with 0.0, 2.5, 5.0, 10.0, or 20.0 mmol/L DADA or DCA, and the concentration of lactic acid in the supernatant was determined after 48 h. As shown in Figure 4A, the lactic acid concentration decreased in a concentration-dependent manner in both DADA- and DCA-treated cells. Using a concentration of 10 mmol/L, the lactic acid concentration of DADA-treated cells was 20.7 ± 1.2 mmol/L, which was significantly lower than that in DCA-treated cells (28.9 ± 0.9 mmol/L) (*P* < 0.05) This difference was more significant when the concentrations of DADA and DCA were increased to 20 mmol/L (*P* < 0.01). Cells were incubated with 10 mM DADA and DCA to determine the time effect on lactic acid production. As shown in Figure 4B, the acetic acid concentration of DADA-treated cells was lower than that of DCA-treated cells, and this difference was significant after 48 h.

Glucose consumption in MDA-MB-231 cells was also evaluated. Glucose consumption decreased after DCA and DADA treatment in a concentration-dependent manner. The glucose consumption of cells treated with 10mM DADA decreased more significantly than cells treated with 10mM DCA (Figure 4C). The time course assay also revealed that the glucose consumption of DADA-treated cells was significantly less than that of DCA-treated cells after 48-h treatment (Figure 4D). Taken together, DADA changed the energetic metabolism of MDA-MB-231 cells, and its effect was stronger than that of DCA.

### DADA induces complete autophagy in early stages of treatment and incomplete autophagy in late stages of treatment

**Figure 5:**
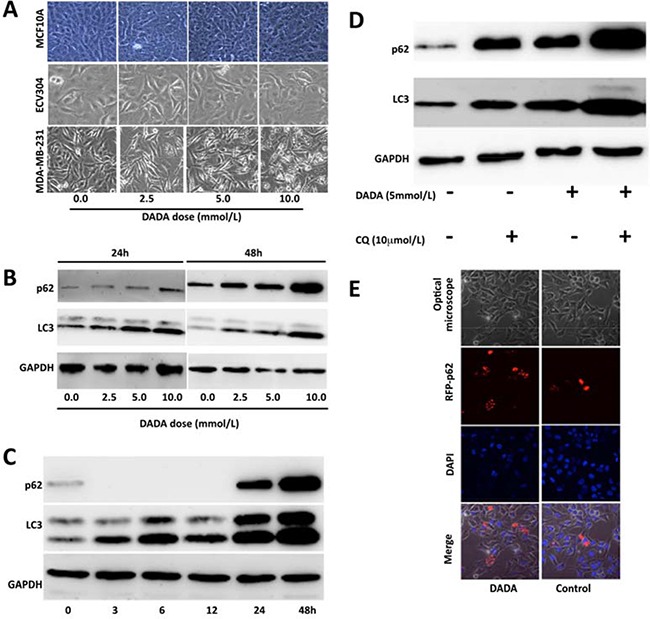
DADA induces autophagy **A.** MDA-MB-231 cells and two non-tumorigenic epithelial cell lines were treated with 0.0, 2.5, 5.0, or 10 mM DADA for 24 h. Cell morphology was then examined using an optical microscope. **B.** MDA-MB-231 breast cancer cells were treated with 0.0, 2.5, 5.0, or 10 mM DADA for 24 or 48 h before p62 and microtubule-associated protein 1 light chain 3 (LC3) levels were determined by Western blot. **C.** Time course of p62 and LC3 levels. The DADA concentration was set at 10 mM, and the expression of p62 and LC3 was evaluated after 0, 3, 6, 12, 24, and 48 hours. **D.** Cells were incubated with or without 5 mM DADA for 19 h before incubating with or without 10 μM/L chloroquine for an additional 5 h. The p62 and LC3 levels were then examined. **E.** Cells were transfected with a plasmid encoding red fluorescent protein and p62 after incubation with or without 10 mmol/L DADA for 24 h. The location relationship between p62 and vacuoles was examined by confocal microscopy. RFP, red fluorescence protein.

Other metabolism-related processes, including autophagy, are altered in cancer cells. Cancer cells modulate autophagy to fight against an unfavorable microenvironment and promote cell survival. However, continued and excessive autophagy can lead to autophagic cell death. Previous studies found that DCA can inhibit tumor growth via autophagy induction [26, 27]. To determine the role of autophagy, MDA-MB-231 cells and two non-tumorigenic epithelial cell lines (MCF 10A and ECV304) were treated with 0.0, 2.5, 5.0, or 10 mmol/L DADA for 24 h. The cells were observed under an optical microscope, and unequal-sized vacuoles were observed in a concentration-dependent manner in treated cells (Figure 5A). In contrast, the above cellular pathological characteristics were not observed in the two non-tumorigenic epithelial cell lines (Figure 5A).

Next, to evaluate whether the vacuoles were associated with autophagy, we examined p62 and LC3. MDA-MB-231 cells were treated with a final concentration of 0.0, 2.5, 5.0, or 10 mmol/L DADA for 24 or 48 h. The levels of p62 and LC3 in the cells were evaluated by Western blot. As shown in Figure 5B, the levels of p62 and LC3 increased gradually with DADA concentration. When the concentration of DADA reached 10 mmol/L, both the p62 and LC3 levels increased greatly when compared with no treatment (Figure 5B). These results suggest that the vacuoles in the tumor cells were positively correlated with the levels LC3 and p62.

To determine the time course of p62 and LC3 levels, the DADA concentration was set at 10 mmol/L, and the levels of p62 and LC3 were evaluated after 0, 3, 6, 12, 24, and 48 h. As shown in Figure 5C, the LC3 level increased with incubation time, which suggested that persistent autophagy was induced by DADA throughout the treatment. Interestingly, p62 levels were low at the beginning of treatment, but were undetectable after 12 h of treatment; however, the p62 levels increased dramatically after 24 h of treatment (Figure 5C). These results suggest that DADA may induce autophagy, and that cells can degrade the autophagosome during early phases of treatment but cannot degrade the autophagosome at later stages of treatment, which could lead to cell death.

To confirm this possibility, chloroquine, an inhibitor of autophagy, was used in the following experiments. Cells were incubated with or without 5 mmol/L DADA for 19 h, and then incubated with or without 10 μM/L chloroquine for an additional 5 h. The p62 and LC3 levels were then examined. As shown in Figure 5D, the p62 and LC3 levels increased to a greater degree in DADA-treated cells compared with untreated cells after adding chloroquine, suggesting that excessive autophagy occurred in DADA-treated cells.

To observe the intracellular accumulation of p62 during excessive autophagy, and to confirm the cellular location of p62, the cells were transfected with a plasmid encoding red fluorescent protein and p62. The relationship between p62 expression and vacuole formation was examined after treatment with 10mM DADA for 24 h through confocal microscopy. As shown in Figure 5E, no vacuoles were observed in control cells, and the level of red fluorescent protein was lower compared with DADA-treated cells. In contrast, vacuoles were seen in DADA-treated cells and the level of red fluorescence was higher, confirming that p62 was located in the autophagosome membrane.

## DISCUSSION

As an inhibitor of the 3-phosphoinositide dependent protein kinase 1 (PDPK1)–4 family, DCA reportedly inhibits tumor proliferation by reversing the bio-energetic profile of cancer cells [26, 27]. A study on the safety and efficacy of DCA in glioblastoma and other recurrent brain tumors is ongoing (https://clinicaltrials.gov/show/NCT01111097). As a derivative of DCA, DADA or C6-H15-N.C2-H2-Cl2-O2, composed of diisopropylamine and DCA, is an over-the-counter drug used to treat nonalcoholic fatty liver disease [28]. However, few studies have examined its potential for treating breast cancer.

In this report, our *in vitro* data showed that the IC_50_ of DADA against the MDA-MB-231 cell line was 7.1 ± 1.1 mmol/L, which was significantly lower than that of DCA (15.6 ± 2.0 mmol/L). In addition, no cytotoxicity was observed in ECV304 and MCF 10A non-tumor cell lines, even using a 20mM concentration. Secondly, *in vivo* experiments showed that a 100 mg/kg (0.0004 mol/kg) DADA gavage inhibited tumor growth to a greater degree than 100 mg/kg (0.0008 mol/kg) DCA, suggesting that DADA has a greater potential for treating cancer through oral administration. Histological examination also showed that DADA and DCA induced tumor necrosis, inflammation, and fibrosis, and that DADA may inhibit Ki67 expression, suggesting that DADA may inhibit mitosis. The effect of DADA on MDA-MB-231 cell metabolism was greater than that of DCA, and the levels of lactic acid production and glucose uptake were inhibited to a greater degree by DADA compared with DCA. Results from autophagy studies also revealed that DADA may induce complete autophagy in earlier stages of treatment, and incomplete autophagy and cell death in later stages of treatment, which may be one of its major anti-cancer effects.

Previous reports have demonstrated that DCA has anti-cancer effects due to its ability to inhibit pyruvate dehydrogenase, reverse glycolysis, and increase oxidative phosphorylation in cancer cells [26, 27, 29–33]. However, these effects are still disputed, and opposing results have been found in studies using colorectal cancer models [27]. For breast cancer, *in vitro* experiments performed on human breast epithelial carcinoma cells (T-47D) showed that the combination of DCA and arsenic trioxide was more effective at inhibiting cell proliferation and inducing cell death than either drug alone [34]. *In vivo*, DCA treatment resulted in a 58% reduction in the number of lung metastases after injection of 13762 MAT rat mammary adenocarcinoma cells [35]. Our data showed that DCA and DADA both possessed anticancer capacities, and had *in vitro* and *in vivo* anti-cancer effects on MDA-MB-231 cells.

Although we had not evaluated the effects of DADA on pyruvate dehydrogenase activity, our data that DADA inhibits lactic acid production and reduces glucose uptake in MDA-MB-231 cells reflected the effects of DADA on pyruvate dehydrogenase activity indirectly. Regarding to the difference in the efficacy of DADA and DCA on the basis of pyruvate dehydrogenase inhibitory activities, the molecular radicals, diisopropylamine in DADA molecule, should play a key role in this difference.

Cells maintain a basal level of autophagy to maintain cell homeostasis [36]. Autophagy is increased to recycle damaged cellular organelles and excess metabolites when cells are exposed to hypoxia, nutrient starvation, growth factor deficits, endoplasmic reticulum stress, accumulation of abnormal products, or organelle injury [36, 37]. Tumor cells can take advantage of this mechanism to survive [36]. However, recent research shows that autophagy may also suppress cancer growth. Lin et al. demonstrated that DCA induced autophagy in colorectal cancer cells, and that DCA treatment decreased pS6 ribosomal protein and translation repressor protein (p4E-BP1) expression, and increased monocarboxylate transporter (MCT)-1 and autophagy [26].

In this study, the time course experiments revealed that p62 levels decreased during the first 3–12 h of treatment, but then increased after 24–48 h. These results suggest that DADA induces complete autophagy during the early phases of treatment and incomplete autophagy during the later stages of treatment, and that a high dose of DADA may result in cell death. Together, the results suggest that three main mechanisms may be responsible for the anti-tumor effects of DADA. First, DADA may suppress ki67 expression and inhibit cancer cell proliferation. Second, DADA could reverse energy metabolism in cancer cells, and third, DADA could induce excessive autophagy and death in cancer cells.

Cell death is a prominent feature of malignant tumors, and occurs in three forms: apoptosis, necroptosis, and autophagy [17, 38]. A recent report by Lin et al. showed that DCA treatment induced autophagy in HT29 tumors with minimal apoptosis [26]. Necroptosis is associated with unprogrammed cell death resulting from cellular damage or pathogen infection [38]. Together, these results suggest that cell death plays a role in the anti-cancer effect of DADA.

DADA has been used as an over-the-counter drug for decades; thus, its safety has been proven [28, 39]. TOXNET indicates that DADA can stimulate smooth muscle and strongly inhibit acetylcholinesterase activity (http://chem.sis.nlm.nih.gov/chemidplus/rn/660-27-5). The median lethal dose (LD50) of oral treatment in mouse is as high as 1700 mg/kg [40]. In this report, 100 mg/kg DADA inhibits tumor growth significantly. Our in vitro data shows that, no cytotoxicity was observed in ECV304 and MCF 10A non-tumor cell lines, even using a 20mM concentration (Figure 5A). In addition, a recent study examined DCA and DADA toxicity, and found that mice implanted with 0.864g DCA or 1.635g DADA-loaded electrospun mats under the subcutaneous layer produce minimal extra physiological toxicity at doses with optimal anti-cancer activity [27]. In conclusion, this study provides evidence for the potential application of DADA in the treatment of breast cancer.

## MATERIALS AND METHODS

### Ethics

This study was approved by the Ethics Committee on Animal Experimentation of Nanjing University. All animal experiments were conducted according to the updated Guide for the Care and Use of Laboratory Animals, Institute for Laboratory Animal Research, Division on Earth and Life Studies, National Research Council of the National Academies (Washington, D.C.).

### Chemical compounds

Sodium DCA (Molecular Formula: C2H2Cl2O2; Molecular Weight: 128.94 g/mol) was purchased from Wako Pure Chemical Industries (Osaka, Japan) and DADA (Molecular Formula: C6-H15-N.C2-H2-Cl2-O2; Molecular Weight: 230.13 g/mol) was purchased from Zhejiang J&C Biological Technology Co., Limited (Zhejiang, China).

### Cell lines

MDA-MB-231 (ATCC® HTB-26™) cells, a breast adenocarcinoma cell line isolated from the pleural effusion of a 51-year-old Caucasian female, were cultured according to American Type Culture Collection (ATCC) guidelines. The base medium for this cell line was ATCC-formulated Leibovitz's L-15 Medium, Catalog No. 30-2008 (ATCC, Manassas, VA). Fetal bovine serum (ATCC, Manassas, VA) was added to a final concentration of 10% to the base medium to make complete growth medium. MDA-MB-231 cells were cultured at 37°C in an incubator with free gas exchange and atmospheric air. Other cell lines used in the preliminary experiments, including MHCC-97H, BEL-7402, MHCC-LM3, QBC939, PANC-1, SGC-7901, and MCF7, were maintained in the conditions suggested by the providers.

### 3-(4,5-dimethylthiazol- 2-yl)-2,5-diphenyl-tetrazolium bromide (MTT) assay

The half maximal inhibitory concentration (IC_50_) of DADA and DCA against cancer cell proliferation was measured by MTT assay. MDA-MB-231 cells were seeded at a density of 1,000 per well in a 96-well plate; after adhering for 24 h, DADA and DCA solutions at final concentrations of 128.00 mmol/L to 0.25 mmol/L were added to the cells in the 96-well plate. After 48 h of incubation, 20 μL of MTT reagent (Vybrant® MTT Cell Proliferation Assay Kit; Thermo Fisher, Nanjing, China) was added to each well and incubated at 37°C for 3 h. After removing the supernatant, 100 μL of isopropanol was added to each well before shaking for 10 min. Absorbance was then measured at 570 nm using a spectrophotometer. The IC_50_ values were calculated using the following formula: lgIC_50_ = Xm − I (P − (3 – Pm − Pn)/4). The Xm is the maximum dose, I is the maximum dose/adjacent dose, P is the positive reaction rate, Pm is the largest positive reaction rate, and Pn is the minimum positive reaction rate.

### Energy metabolism targeting analysis

To evaluate the effect of DCA and DADA on energy metabolism, lactic acid production and glucose uptake were measured in MDA-MB-231 cells. The cells were treated with 0.0 to 20.0 mmol/L DADA or DCA, and lactic acid and glucose concentrations were measured after 48 h using a Lactic Acid Test Kit (ChemWorld, Kennesaw, GA) and a Glucose Assay Kit (Abcam, Cambridge, MA). To observe the time course of DCA or DADA treatment on lactic acid production and glucose uptake, DADA or DCA concentrations were set at 10 mmol/L, and lactic acid and glucose concentrations were measured at 6, 12, 24, and 48 h.

### Subcutaneous transplantation tumor model

Six- to 8-week-old female nude BALB/c mice (16.8 ± 0.2 g) were purchased from the Animal Research Institute of Nanjing University, Nanjing, China. An experimental breast carcinoma was created *in vivo* by subcutaneous transplantation of 5 × 10^6^ MDA-MB-231 cells (suspended in 100 μL of 10% Matrigel®, BD Biosciences, Nanjing, China) into the right rear flanks of BALB/c mice. Tumor growth was observed every day, and tumor volume was calculated by the formula: volume = length × width^2^/2. When the tumor volume reached 100–150mm^3^, the mice were divided into control, DCA, and DADA groups (n = 5 in each group). The mice groups were lavaged once a day with 100 μL 0.9% saline, 100 mg/kg DADA (0.0004 mol/kg), and 100 mg/kg DCA (0.0008 mol/kg) for 24 d. Tumor volumes were measured every 3 d, and isolated and weighed after 24 d.

### Histological examination

After 24 d, all tumors were isolated for histopathological examination. Following fixation of the tumor samples in 10% formalin in phosphate buffer solution (PBS), sections (4–5-μm thick) of the tumor tissues were stained by hematoxylin and eosin (H&E). Ki67 is a nuclear antigen associated with cell proliferation, and its function is closely related to mitosis. To study the expression of Ki67, immunohistochemical staining was performed on tumor sections. Endogenous peroxidase activity was blocked by 3% H_2_O_2_ for 10 min at room temperature. After rinsing three times with PBS, an anti-Ki67 rabbit polyclonal antibody (1:200; Biovisualab, Shanghai, China) diluted in 0.1% PBS was added to the sections and incubated for 2 h at 4°C. The slides were rinsed with 0.1% PBS three times before adding the biotin-labeled rat anti-rabbit antibody (1:200; Biovisualab, Shanghai, China) diluted in 0.1% PBS and incubating at room temperature for 20 min. After rinsing three times, chromogenic detection was performed using 3,3′-diaminobenzidine (DAB) (Thermo Fisher, Nanjing, China). Histological examinations were conducted and photographs were taken using a NanoZoomer 2.0-RS optical microscope (Hamamatsu Photonics, Hamamatsu, Japan).

### Autophagy assay

To determine the role of autophagy following DADA treatment, MDA-MB-231 breast cancer cells and two non-tumorigenic epithelial cell lines, MCF 10A and ECV304, were treated with 0.0, 2.5, 5.0, or 10 mmol/L DADA for 24 h. Autophagy was first observed under an optical microscope. To evaluate whether autophagy was altered by DADA in a concentration-dependent manner, MDA-MB-231 breast cancer cells were treated with 0.0, 2.5, 5.0, or 10 mmol/L DADA for 24 or 48 h. The levels of two autophagic markers, autophagy degradation receptor (p62) and microtubule-associated protein1 light chain 3 (LC3), were detected by Western blot. Primary antibodies against p62 and LC3 were purchased from Biovisualab, Shanghai, China. To determine the time course of autophagy, cells were incubated with 10 mmol/L DADA, and p62 and LC3 levels were evaluated after 0, 3, 6, 12, 24, and 48 h. To prove that incomplete autophagy occurred, cells were incubated with or without 5 mmol/L DADA for 19 h and then incubated with or without 10 μM/L chloroquine (Sigma-Aldrich, Nanjing, China) for an additional 5 h. The p62 and LC3 levels were evaluated by Western blot. To confirm the position of p62, MDA-MB-231 cells were transfected with a plasmid encoded with red fluorescent protein and p62, after incubation with or without 10 mmol/L DADA for 24 h, the cellular location of p62 and vacuoles were checked using confocal laser scanning microscopy (Nikon, Tokyo, Japan).

### Statistical analysis

Data are shown as the mean ± standard deviation (SD). Differences among groups were examined using a Kruskal–Wallis H test or a one-way analysis of variance (ANOVA). The level of statistical significance was set at 0.05. Statistical analyses were performed using SPSS 19.0 for Windows (SPSS, Inc., Chicago, IL).
